# Management of sequelae of septic arthritis of hip

**DOI:** 10.4103/0019-5413.37008

**Published:** 2007

**Authors:** KL Jagadishwer Rao, Durga Prasad, Kantilal Jain

**Affiliations:** Department of Orthopaedics, Princess Esra Hospital, Deccan College of Medical Sciences, Shah Ali Bhanda, Hyderabad - 500 065, AP

**Keywords:** Sequelae of septic arthritis of hip, pelvic osteotomy, reconstruction of hip

## Abstract

A nine years old boy, who had suffered septic arthritis at the age of two years and presented now with a limp, hip instability, leg length discrepancy. The patient was treated by adductor tenotomy and upper tibial pin traction. When head remnant reached the level of the acetabulum, open reduction and Pemberton osteotomy was done to achieve cover of the femoral head. The purpose of this report is to highlight the six years followup of reconstruction of sequale of septic arthritis of hip joint.

The ultimate goal of the management of sequelae of septic arthritis of the hip centers around stabilizing the hip with minimal loss of movement, thereby improving the gait. The outcome of the treatment for septic arthritis of hip depends on the age of the patient and clinico-radiological status of the hip. Choi's^1^ radiological classification is being used in the surgical management of the sequelae of septic arthritis of the hip. We are presenting a case of a boy aged nine years, with coxa breva, deformed neck and pathological dislocation of about seven years duration. Treating the sequelae at this stage of the disease is quite challenging, hence the presentation.

## CASE REPORT

**Figure 1:**
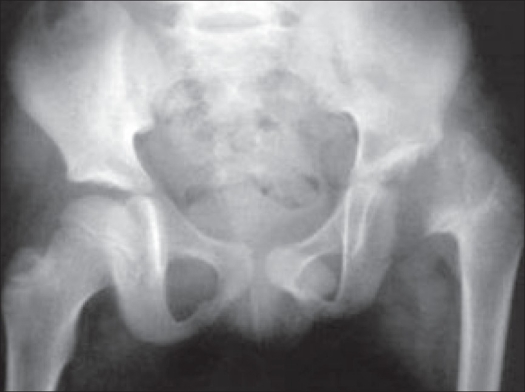
X-ray of the pelvis showing coxa breva with subluxed remnant of head of femur with deformed and bifurcated neck

A boy aged nine years complained of painless limp on the left side and came walking on his tiptoes. He had suffered septic arthritis of left hip at the age of two years. On examination, there was muscle wasting around the hip and thigh, with no fullness in the Scarpa's triangle. Greater trochanter was proximally migrated. The patient had Trendelenberg gait, with fixed flexion and adduction deformity of 30°, no rotational deformity and shortening of 4cm. Further flexion of the hip was possible up to 120°. No abduction or rotations were possible. The telescopy was neither appreciable nor demonstrable. Skiagram of the pelvis revealed coxa breva with subluxated head of femur with deformed and bifurcated neck [Figure 1].

Magnetic resonance imaging (MRI) was not done as the patient could not afford it.

**Figure 2:**
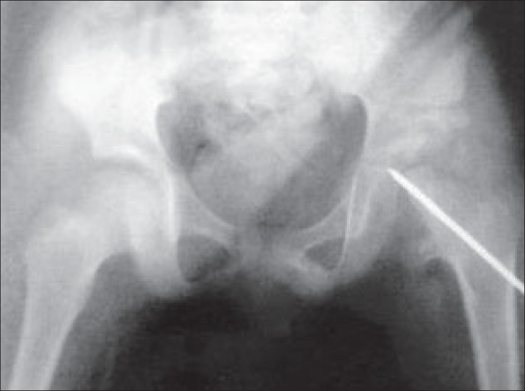
X-ray eight weeks after surgery shows reduced proximal femur with a pin

**Figure 3:**
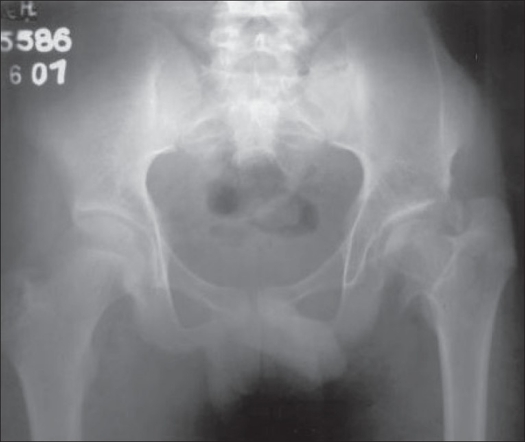
X-ray after six years of surgery shows near completely formed head

Initially, open adductor tenotomy was done and tibial pin traction was applied for 21 days, continued till the head or the deformed neck could be brought to the level of the acetabulum. The open reduction of the hip was performed through anterior illio-femoral approach using a bikini incision. We found a small head and bifurcated neck and soft tissue occupying the medial acetabulum. We could reduce the head into the acetabulum, which was quite stable. Pemberton's osteotomy, also offered a good lateral cover to the head. A subcutaneous K-wire was passed across the hip joint from the trochanter [Figure 2]. Hip spica was given for 45 days. As the hip was stable, the patient was able to walk after eight weeks supervised physiotherapy which included gait training and active and passive hip and knee exercises to gain hip movements. The patient was followed up for six years [Figure 3] and in this period the head had increased in size, hip was stable, with 2 cm, shortening. The abduction was restricted to 20° and adduction to 10°. The patient was able to squat with little difficulty.

## DISCUSSION

The involvement of hip in septic arthritis is particularly difficult to detect. It is often diagnosed late, leading to irreversible damage to the articular cartilage, blood supply to the epiphysis and sometimes absorption of head and neck, resulting in severe shortening and disability. Zwierzchovoski^4^ analyzed the sequelae of septic arthritis of hip.

Different procedures have been described for different stages of sequelae of septic arthritis of hip. All procedures advocated for the management of septic arthritis of hip grouped under Choi type I-IV, have variable results. Some of the suggested surgical treatment for children according to Choi's classification is:

Type I: Almost normal hip or mild coxa magna. It needs no reconstruction.

Type II: Deformed epiphysis, physis, metaphysis may result in coxa breva or progressive coxa vara or coxa valgus. It needs surgical intervention to prevent subluxation.

Type III: Malalignment of femoral neck, excessive anteversion or retroversion with pseudoarthrosis. It neccesitates a realignment surgery for proximal femur or bone grafting for Pseudo-arthrosis.

Type IV: Destruction of the head and neck of femur with the presence of remnant of medial base of neck. Complex clinical problems with limb length inequality -needs reconstructive surgery.^3^,^5^,^6^

Our patient was grouped under Choi's^1^ Type IV with doubtful small head, bifurcated neck and pathological dislocation [Figure 1]; we treated it on lines similar to DDH.

Adductor tenotomy with skeletal traction before performing an open reduction and Pemberton's osteotomy helps in bringing the greater trochanter down to the level of the hip. We chose Pemberton's osteotomy to cover the head and gain some length. Pemberton's and Dega's osteotomy^7^ are indicated when the head is small, easily reducible. These incomplete osteotomies (done in shortening of limbs) tend to exert more of lengthening effect than does Salter's osteotomy.^8^ All the suggested surgical treatment must be regarded as measures that temporarily improve the clinical function and delay the more definite procedures that are reserved for adult patients. In our case, we could give the patient's femoral head a fair chance to remodel on a followup till the age of 15 years. The femoral head has increased in size with stable hip and 2 cm shortening. The abduction was restricted by 20°, adduction by 10°, extension was 0° and the patient was able to squat with little difficulty. With a 2 cm shoe raise, the patient had no limp and he went back to his school [Figure 3]. In the event of functioning complications like Avascular necrosis (AVN), the patient can wait till he is fit to undergo THR at a later stage.

Our procedure has given a satisfactory acceptable function of hip in a Choi type IV sequelae of septic arthritis of hip below 10-12 years of age.
